# Translation of the Modified Polycystic Ovary Syndrome Questionnaire (mPCOSQ) and the Polycystic Ovary Syndrome Quality of Life Tool (PCOSQOL) in Dutch and Flemish Women with PCOS

**DOI:** 10.3390/jcm12123927

**Published:** 2023-06-08

**Authors:** Geranne Jiskoot, Sara Somers, Chloë De Roo, Dominic Stoop, Joop Laven

**Affiliations:** 1 Division of Reproductive Endocrinology and Infertility, Department of Obstetrics and Gynaecology, Erasmus Medical Centre, P.O. Box 2040, 3000 CA Rotterdam, The Netherlands; 2 Department of Reproductive Medicine, Ghent University Hospital, Corneel Heymanslaan 10, 9000 Ghent, Belgium

**Keywords:** polycystic ovary syndrome, PCOS, health-related quality of life, QoL, questionnaire, modified PCOSQ, PCOSQOL

## Abstract

This study aims to determine the test–retest reliability and to confirm the domain structures of the Dutch version of the modified polycystic ovary syndrome questionnaire (mPCOSQ) and the Polycystic Ovary Syndrome Quality of Life Scale (PCOSQOL) in Dutch and Flemish women with Polycystic Ovary Syndrome (PCOS). PCOS patients were contacted with a request to complete both questionnaires (including additional demographic questions) online in their home environment on T0 and on T1. The study was approved by the Ethics Committee of Erasmus Medical Centre and of Ghent University Hospital. In this study, 245 participants were included between January and December 2021. The mPCOSQ has excellent internal consistency (α: 0.95) and a high to excellent Intraclass Correlation Coefficient (ICC) for all six domains (ICC: 0.88–0.96). The PCOSQOL demonstrates excellent internal consistency (α: 0.96) and ICC (ICC: 0.91–0.96) for all four domains. The original six-factor structure of the mPCOSQ is partly confirmed. An extra domain is added to the PCOSQOL which included coping items. Most women have no preference for one of the two questionnaires (55.9%). In conclusion, The Dutch mPCOSQ and PCOSQOL are reliable and disease-specific QoL measures for women with PCOS. Both questionnaires are recommended for clinical practice.

## 1. Introduction

Polycystic Ovary Syndrome (PCOS) is the most common endocrine condition in women of reproductive age and affects 8–15% of that age group [1,2,3]. The diagnosis of PCOS requires at least two out of three of the following criteria: (1) oligo-ovulation or anovulation (irregular or no menstrual cycle), (2) clinical hyperandrogenism (e.g., hirsutism) and/or biochemical signs of hyperandrogenism (elevated free androgen index or elevated testosterone levels), and (3) polycystic ovarian morphology (PCOM) (by transvaginal ultrasound), and the exclusion of other etiologies that might cause hyperandrogenism or cycle irregularity [4]. Most women with PCOS experience one or more of the following symptoms: psychological (anxiety, depression, and body image dissatisfaction), physical (hirsutism and acne), reproductive (irregular menstrual cycles, and infertility), and metabolic (insulin resistance, metabolic syndrome, prediabetes, and type 2 diabetes) disorders [5]. In general, women with PCOS encounter more depressive and anxiety complaints, they have lower self-esteem, and they experience a more negative body image than women without PCOS [6,7]. The prevalence of clinically significant symptoms associated with depression among women diagnosed with PCOS is reported to be 37%, which is notably higher compared to the rate of 14.2% observed in healthy women. Furthermore, the prevalence of symptoms related to anxiety is found to be 42% in women with PCOS, whereas it is only 8.5% among healthy women [8]. Additionally, these women report lower quality of life (QoL) due to their PCOS symptoms [9,10]. Most women with PCOS report that weight concerns have the largest impact on QoL. Other PCOS symptoms, such as menstrual problems, infertility, hirsutism, and acne, impact their QoL less [11]. According to the latest PCOS guideline of the European Society of Human Reproduction and Embryology (ESHRE), healthcare professionals should be more aware of the negative impact of PCOS on quality of life [12].

Implementing QoL questionnaires in research and routine practice provides significant added value in understanding the impact of a particular condition or treatment on an individual’s well-being and overall quality of life. QoL questionnaires are standardized instruments that assess various aspects of an individual’s physical, psychological, and social functioning, and their overall satisfaction with life. For women with PCOS, the polycystic ovary syndrome questionnaire (PCOSQ) [13] was developed to complement generic health-related QoL instruments, such as the Standard short-form health survey questionnaire (SF-36) [14]. The PCOSQ has already been translated into English [15,16], Arabic [17], German [18], Chinese [19], Swedish [20], and Iranian [21]. Recent research suggested that psychological, social, or environmental aspects are less represented than the physical impact of PCOS measured by the PCOSQ. Others suggested that more QoL measures should be developed [22] to obtain a more sensitive measure of QoL in all PCOS phenotypes [23]. Therefore, the Polycystic Ovary Syndrome Quality of Life Scale (PCOSQOL) was developed in 2018 based on these recommendations [24].

Globally, women have reported insufficient access to information, delayed diagnosis, and inconsistent care for PCOS [12]. The provision of comprehensive and accurate information has been shown to enhance satisfaction with care and improve the overall patient experience [25]. Language barriers further exacerbate the issue, as the majority of consumer information is predominantly available in English. This poses challenges for immigrant populations and women residing in countries where English is not their primary language, such as the Netherlands and Belgium. Therefore, this study aims to determine the test–retest reliability and to confirm the domain structure of the Dutch version of the mPCOSQ and the PCOSQOL in Dutch-speaking samples. Additionally, we want to examine which questionnaire is preferred by women with PCOS.

## 2. Materials and Methods

### 2.1. Study Design

Two independent translators performed a forward and backward translation of the original English mPCOSQ and PCOSQOL. Between January and December 2021, patients with PCOS were contacted with a request to complete the mPCOSQ and PCOSQOL questionnaires (and some additional demographic questions) online in their home environment (T0). A test–retest design was applied to demonstrate stability over time by having all women complete the same questionnaires a second time after two to four weeks (T1). At both points in time, participants were asked if they had a preference for one of the two questionnaires. We also performed a factor analysis. All materials, including the information sheet, the consent form, and the questionnaires, were completed using Gemstracker (www.gemstracker.org). The study was approved by the Ethics Committee of the Erasmus Medical Centre (The Netherlands) (MEC-2019-0628) and Ghent University Hospital (Belgium) (B6702020000388).

### 2.2. Questionnaires

The original PCOSQ [13] was developed in 1998 by Cronin et al. It consisted of 26 items and took 10 to 15 min to complete. The questionnaire included five subscales: emotions, body hair, infertility, weight, and menstrual problems [13]. Based on a validation study by Jones and colleagues [15], an acne domain was added to improve the validity of the PCOSQ. This resulted in a 30-item mPCOSQ with 6 subscales: emotions (8 items), body hair (5 items), weight concerns (5 items), infertility concerns (4 items), menstrual problems (4 items), and acne (4 items). Each item is answered based on a 7-point Likert scale, where one represents the poorest function, and seven represents an optimal function. A higher score on the mPCOSQ denotes a higher quality of life [26]. The PCOSQOL is developed by Williams et al. and is a 35-item self-administered questionnaire with four domains: impact of PCOS (16 items), infertility (7 items), hirsutism (6 items), and mood (6 items). Each item is answered based on a 7-point Likert scale, ranging from 1 (usually) to 7 (does not apply). A higher score on the PCOSQOL denotes a higher quality of life [24].

### 2.3. Population

Women of at least 18 years old, who were able to speak and write Dutch, and who were diagnosed with PCOS according to the Rotterdam criteria [4] or according to the international evidence-based guideline for the assessment and management of PCOS [12] were eligible for the study. Women who were pregnant at T0 or T1 were excluded from the study. The definition of PCOM varies according to the PCOS criteria that are used. According to the Rotterdam criteria, PCOM is defined as ≥12 follicles (measuring 2–10 mm) and/or ovarian volume > 10 cm^3^ in at least one ovary [27]. According to the international evidence-based guideline for the assessment and management of PCOS, PCOM is defined as a follicle number per ovary of ≥20 (on either ovary) and/or an ovarian volume ≥ 10 mL, ensuring no corpora lutea, cysts, or dominant follicles [12]. Both definitions are used to define PCOS in this study.

### 2.4. Recruitment

Participants were recruited via Erasmus MC (the Netherlands) and Ghent University Hospital (Belgium). At the Erasmus MC, all patients were recruited via a PCOS database of the Division of Reproductive Endocrinology and Infertility. This database includes all women with menstrual cycle disorders that are systematically screened using a standardized protocol. They were all contacted via email and received a link to the online questionnaires. At Ghent University Hospital, Belgian PCOS patients were recruited in several ways: (1) PCOS patients who were included in a previous study and who agreed to be contacted for future research received an email with the link to the questionnaires, (2) the gynecologists working at the Women’s Clinic and in a later stage the endocrinologists working at the Endocrinology Department of the hospital were informed about the study and recruited patients in their daily practice, (3) the treating gynecologists of the Department of Reproductive Medicine (RM) emailed PCOS patients that were eligible for the study with a request to reply in case of interest in the study, (4) a flyer was distributed in the waiting room of the Department of RM and later in the waiting room of the Endocrinology Department of the hospital, (5) a message was put on the website of the Department of RM. In May 2022, it was decided to stop recruitment. At that time, 64 Belgian PCOS patients had completed the questionnaires at both points in time.

### 2.5. Statistical Considerations

Applying the procedure described by Bonett [28] for a Pearson correlation of at least 0.80 and a 95% confidence interval width of 0.10, requires a sample size of 240 patients. Therefore, 120 Dutch patients and 120 Flemish patients need to be enrolled.

The Cronbach alpha was used together with the Intraclass Correlation Coefficient (ICC) for the test–retest of the mPCOSQ and the PCOSQOL. To assess the reliability within the mPCOSQ and the PCOSQOL domains, the one-way random-effects analysis of variance technique was used to estimate the Mean Square values required for subsequent calculation of the ICC. The ICC ranges from 0 to 1. Values near 0 indicate unreliable test–retest structure, and values above 0.90 indicate excellent reliability. Factor analysis (using principal components analysis with varimax rotation) was used to measure which questions belong to each domain. The factor indicates the relationship between a set of items and is defined by the items that load on it or the factor loadings. Loadings > 0.5 were considered satisfactory. The data were analyzed using SPSS Software version 28 (IBM). Data not normally distributed were presented as medians, including the interquartile range (IQR) for continuous data and n (%) for categorical data. Mann–Whitney U-tests were used to determine significant differences between continuous variables. For categorical variables, Fisher’s exact and χ2 tests were used. *p*-values of ≤0.05 defined statistical significance.

## 3. Results

A total of 245 women participated in the study and completed the test–retest of both questionnaires; 64 women were included in Belgium and 181 in the Netherlands. The median age of the women who completed the mPCOSQ and the PCOSQOL assessments was 31 (IQR 27.0–34.0) years. The median weight was 79 (IQR 65.0–98.0) kg. The median BMI was 32.1 (IQR 27.7–39.2) kg/m^2^. Most women received their PCOS diagnosis one to five years ago (44.1%) and were not actively trying to become pregnant (63.7%). Most women were married (40.8%) and worked on a full-time basis (45.3%). They did not smoke (91.4%), drank alcohol (58.4%), and the majority did not use drugs (98.0%). Most women were Caucasian and were born in the Netherlands or in Belgium (89.0%). Seven women in total were born in Suriname (3.3%), Turkey (0.8%), and Morocco (0.4%), and fourteen women were born elsewhere (5.7%), see Table 1. At baseline, women in Belgium had a significantly lower BMI (*p* = 0.003) and were more likely to work on a full-time basis (*p* < 0.001). There were no significant differences in age (*p* = 0.966), marital status (*p* = 0.141), smoking status (*p* = 0.298), alcohol use (*p* = 0.464), and the proportion of women that were trying to conceive (*p* = 0.291).

### 3.1. Test–Retest Reliability

For the 30-item mPCOSQ, the overall Cronbach’s alpha (α) was 0.95, which is considered excellent internal consistency. The ICC for the six domains ranged from 0.88 to 0.96, which is high to excellent. For the 35-item PCOSQOL, the overall Cronbach’s α was 0.96. The ICC for the four domains ranged from 0.91 to 0.96. These values are considered excellent (Table 2).

### 3.2. Factor Analysis

For the mPCOSQ, we found that the original six-factor structure was partly confirmed (Supplemental Tables S1 and S2). Two items (“How much of the time during the last two weeks did you feel a lack of control over the situation with PCOS?” (item 23) and “In relation to your last menstruation, how much was a late menstrual period a problem for you?” (item 20)) did not load on their original domain. Therefore, item 23 was moved from the domain “infertility” to “emotions”, and item 20 was moved from the domain “emotions” to “menstrual problems”.

For the PCOSQOL, the original four-factor structure was changed into a five-factor structure: impact of PCOS (11 items), infertility (7 items), hirsutism (7 items), coping with PCOS (6 items), and mood (4 items). The domain “coping with PCOS” was added, which includes items from the original domain “impact of PCOS”: 19, 27, 28, 29, 32, and 33. Item 19 (“Felt like your PCOS is in control of your life”) loaded on two domains which are “impact of PCOS” and “coping with PCOS”. We decided to include item 19 in the new domain, “coping with PCOS”. Item 20 (“Felt embarrassed about the way you look”), originally in the domain “hirsutism”, failed to obtain a value of 0.50.

### 3.3. Baseline Differences

The overall total score on the mPCOSQ was 3.97 (SD = 1.20). The difference between the overall score in Belgium and in the Netherlands was significant (4.74 vs. 3.70; *p* <0.001). On the PCOSQOL, women recruited in Belgium had an overall total score of 4.90 (SD = 1.23), and women recruited in the Netherlands had a total score of 3.86 (SD = 1.23) (*p* < 0.001). This suggests that women in Belgium had better QoL compared to women in the Netherlands. The mean scale scores of the mPCOSQ showed that “menstrual problems” and “weight” scored the lowest, indicating the worst health in these two dimensions. For the PCOSQOL, the scales “mood” and “impact of PCOS” scored the lowest (Table 3). Additional analyses were performed to examine the difference between overall scores for Belgium and the Netherlands.

Assuming that BMI performed an important role in the difference between mPCOSQ and PCOSQOL scores in both countries, we performed additional analyses based on BMI and if women were trying to conceive. Women with a BMI below 30 had better QoL compared to women with a BMI above 30 on the mPCOSQ (4.48 vs. 3.93, *p* < 0.002) and on the PCOSQOL (4.64 vs. 3.60, *p* < 0.001). Additionally, women who were not trying to conceive had better QoL compared to women who were trying to conceive based on the mPCOSQ (4.38 vs. 3.68, *p* < 0.001) and the PCOSQOL (4.14 vs. 3.68, *p* = 0.002).

### 3.4. Acceptability

Most women (70.6%) completed the questionnaires in approximately 15 min. They found the time spent on the questionnaires to be good (93.9%). Most women experienced the questionnaires as medium relevant (36.3%), while 23.5% found the questionnaires highly relevant, and 24.1% found them not relevant. Most women had no preference for one of the two questionnaires (55.9%).

## 4. Discussion

The results of this study showed that the Dutch mPCOSQ and PCOSQOL are reliable and disease-specific QoL measures for Dutch and Flemish women with PCOS. Both questionnaires had excellent internal consistency and high to excellent ICC for all domains. For the mPCOSQ, the original six-factor structure was partly confirmed. Based on the factor analysis of the PCOSQOL, an extra domain was added, which included coping items. Most women had no preference for one of the two questionnaires. We found that Belgian women with PCOS had better QoL compared to women in the Netherlands. This might be related to the significantly lower BMI in Belgian patients.

### 4.1. mPCOSQ

The original PCOSQ was translated into many languages [15,16,17,18,19,20] but not yet for Dutch-speaking women. Therefore, we have translated the 30-item Dutch version of the modified PCOSQ, including a sixth subscale for acne [21,26]. The acne domain was added because acne is related to a worse quality of life in women with PCOS [15] and because previous validation studies have shown that adding acne questions improved the validity of the original PCOSQ [15,26]. Others have introduced a version of the mPCOSQ with seven domains; the domain “menstrual factor” of the PCOSQ was divided into “menstrual symptoms” and “menstrual predictability” [26]. This change was installed because Guyatt et al. had found that two questions on the menstrual period (item 8 on irregular menstrual period and item 20 on last (late) menstrual period) loaded on a new—at that time undefined—factor of the PCOSQ [15,17]. Other authors suggested a change of item 20 from the emotional domain to the menstrual domain [20,21]. Another possible explanation for the low internal consistency in the menstrual domain of the PCOSQ was the fact that the question on headaches did not fit in that domain [16]. Due to the inconsistency in the literature, we decided to use the mPCOSQ with six domains. Although some earlier studies found a lower internal consistency for the menstrual domain compared to the other PCOSQ domains [15,16,17,18], our data showed an excellent Cronbach’s α [21]. However, we also found that item 20 did not load on the original emotional domain and suggested it be moved to the menstrual domain, which is in line with the findings of other research groups [20,21]. Our results showed that item 23 (lack of control over the situation with PCOS) should be moved from the domain “infertility” to the emotional domain. This finding is in line with the results of previous studies [15,20].

In our study, the time interval between the completion of the questionnaires was two to four weeks. Previous PCOSQ/mPCOSQ studies have used intervals of three to six days [15], seven days [20], five days to two weeks [17], two weeks [21], four weeks [18], and 44 weeks [16]. Jedel and colleagues suggested an interval of three days to limit the impact on health status [20]. Our belief is that a longer time between T0 and T1 would be preferable to prevent participants from recalling their previous answers and reproducing them at T1. However, we acknowledge that a four-week timeframe may be more prone to changes in health compared to shorter intervals [16]. However, our data showed high to excellent test–retest reliability.

### 4.2. PCOSQOL

Our results with regard to internal consistency and test–retest reliability of the PCOSQOL were in line with the development and preliminary validation study [24]. Contrary to that study, we found that six items of the domain “impact of PCOS” loaded on a new domain “coping with PCOS”. Moreover, it can be debated to exclude items 19 and 20 because of very low loadings to the original domain. This, too, is in contrast with the findings of Williams et al. [24].

### 4.3. Strengths, Limitations, and Future Research

The strengths of our study are worth mentioning. In contrast to some previous studies [17,20], we strived for identical settings (the home environment) at the two points in time of completing the questionnaires [15,21,24]. Furthermore, we questioned the same participants twice [16,18]) and not a subgroup of the first cohort [21] or a different cohort [24]. Additionally, it was a strength that two fertility centers in different countries were involved because most studies were monocentric [15,17,18,19,20] or performed within one country [21,24]. Additionally, we included both PCOS patients with and without a wish for a child and patients with and without a recent PCOS diagnosis which improves the generalizability of the results. Nonetheless, the proportion of participants with irregular cycles and infertility might be larger and the proportion of participants with hirsutism and acne might be smaller compared to the general population since we mainly recruited via fertility centers [15,18,21].

Some limitations could be identified. Although much effort was put into recruitment at Ghent University Hospital, the target of 120 Belgian inclusions could not be reached within a reasonable time. However, a predominance of Dutch patients led to the intended sample size of 240 patients. Yet, this might have influenced the results of the study as significant differences between Belgian and Dutch participants were present at baseline, with Belgians having a lower BMI and being more likely to work on a full-time basis. Additionally, the sample is prone to self-selection bias: it is possible that participants were more eager to take part in the study because of more severe symptoms related to PCOS or because of an impaired quality of life [18]. A small cohort of Belgian participants presented themselves to take part in the study (e.g., after reading a message on the hospital website). For these patients, it was not possible to verify the diagnosis of PCOS [21].

More translations of the PCOSQOL in different ethnic populations are necessary to evaluate if adjustments to the original questionnaire are necessary. Future research should also focus on whether differences in quality of life in PCOS patients could be correlated with different PCOS phenotypes.

## 5. Conclusions

The Dutch mPCOSQ and PCOSQOL are reliable and disease-specific QoL measures for Dutch and Flemish women with PCOS. For the mPCOSQ, the original six-factor structure was partly confirmed. Based on the factor analysis of the PCOSQOL, an extra domain was added, which included coping items. Most women had no preference for one of the two questionnaires.

## Figures and Tables

**Table 1:** Patient characteristics.

Characteristics	Belgium (IQR)	The Netherlands (IQR)
	N = 64	N = 181
Median age (years)	31.0 (28.0–33.8)	30.0 (27.0–34.0)
Median weight (kg)	69.0 (58–80.8)	82.0 (70.0–100.0)
Median body mass index (BMI) (kg/m^2^)	30.0 (25.3–35.5)	34.3 (28.7–40.5)
	N (%)	N (%)
Time since PCOS diagnosis:		
<1 year	16 (25.0)	27 (14.9)
1 to 5 years	31 (48.4)	77 (42.4)
5 to 10 years	9 (14.1)	43 (23.8)
>10 years	8 (12.5)	34 (18.8)
Trying to conceive (yes)	27 (42.2)	62 (34.3)
Marital status (married)	21 (32.8)	79 (43.6)
Education:		
Low	12 (18.8)	6 (3.3)
Intermediate	2 (3.1)	84 (46.4)
High	50 (78.1)	91 (50.3)
Working status (full time)	46 (71.9)	65 (35.9)
Smoking (yes)	3 (4.7)	18 (9.9)
Alcohol use (yes)	40 (62.5)	103 (56.9)
Drug use (yes)	1 (1.6)	4 (2.2)

Note: “Low” refers to the International Standard Classification of Education (ISCED) levels 0–2 (early childhood education, primary education, and lower secondary education), “intermediate” refers to ISCED levels 3–4 (upper secondary education, and post-secondary non-tertiary education), and “high” refers to ISCED levels 5–8 (tertiary education, including bachelor’s, master’s, and doctoral degrees). IQR = interquartile range.

**Table 2:** mPCOSQ and PCOSQOL subscales at T0 and T1.

Questionnaire	Domain	Median Score T0 (Min–Max)	Median Score T1 (Min–Max)	Cronbach’s Alpha (α)	ICC (95% CI)
mPCOSQ	Emotions	3.8 (1–7)	4.1 (1–7)	0.919	0.92 (0.90–0.94)
	Body hair	3.8 (1–7)	3.8 (1–7)	0.959	0.96 (0.95–0.97)
	Weight	3.0 (1–7)	2.8 (1–7)	0.955	0.96 (0.94–0.97)
	Infertility	3.8 (1–7)	4.0 (1–7)	0.932	0.93 (0.90–0.95)
	Menstrual problems	3.3 (1–7)	3.3 (1–7)	0.879	0.88 (0.84–0.90)
	Acne	5.8 (1–7)	5.8 (1–7)	0.916	0.92 (0.89–0.94)
PCOSQOL	Impact of PCOS	3.9 (1–7)	3.9 (1–7)	0.952	0.95 (0.94–0.96)
	Infertility	4.6 (1–7)	4.6 (1–7)	0.951	0.95 (0.94–0.96)
	Hirsutism	4.5 (1–7)	4.3 (1–7)	0.961	0.96 (0.95–0.97)
	Mood	4.2 (1–7)	4.2 (1–7)	0.914	0.91 (0.89–0.93)

Note: CI = confidence interval.

**Table 3:** Baseline mPCOSQ and PCOSQOL subscales for Belgium and the Netherlands.

Questionnaire	Domain	Overall Score (SD)	Score for Belgium (SD)	Score for The Netherlands (SD)	*p* Value
mPCOSQ	Emotions	3.99 (1.36)	4.93 (1.37)	3.65 (1.19)	<0.001
	Body hair	4.00 (2.03)	4.92 (2.01)	3.67 (1.95)	<0.001
	Weight	3.59 (2.19)	4.61 (2.10)	3.22 (2.11)	<0.001
	Infertility	3.73 (1.94)	4.37 (2.08)	3.50 (1.84)	0.002
	Menstrual problems	3.41 (1.40)	3.96 (1.67)	3.22 (1.24)	0.003
	Acne	5.17 (1.75)	5.43 (1.65)	5.09 (1.78)	0.167
	Total score (with acne)	3.97 (1.20)	4.74 (1.24)	3.70 (1.07)	<0.001
	Total score (without acne)	3.78 (1.28)	4.63 (1.28)	3.48 (1.14)	<0.001
PCOSQOL	Impact of PCOS	4.00 (1.47)	4.88 (1.47)	3.70 (1.35)	<0.001
	Infertility	4.38 (1.92)	4.74 (1.89)	4.26 (1.92)	0.094
	Hirsutism	4.29 (1.88)	5.13 (1.74)	3.99 (1.84)	<0.001
	Mood	3.99 (1.42)	4.91 (1.17)	3.67 (1.36)	<0.001
	Total score	4.13 (1.31)	4.90 (1.23)	3.86 (1.23)	<0.001

## Data Availability

The data presented in this study are available on request from the corresponding authors.

## Supplementary Materials

The following supporting information can be downloaded at: https://www.mdpi.com/article/10.3390/jcm12123927/s1. Table S1: mPCOSQ factor analysis; Table S2: PCOSQOL factor analysis; File S1: Dutch version of the mPCOSQ; File S2: Dutch version of the PCOSQOL.
