# Supplementary material for: Translation of the Modified Polycystic Ovary Syndrome Questionnaire (mPCOSQ) and the Polycystic Ovary Syndrome Quality of Life Tool (PCOSQOL) in Dutch and Flemish Women with PCOS
Source: J Clin Med. 2023 Jun 8;12(12):3927. doi: 10.3390/jcm12123927 (PMC10299068; doi:10.3390/jcm12123927)
Supplement: Supplementary file 1 [file jcm-12-03927-s001.zip › Supplementary File S1 Dutch version of the mPCOSQ.pdf]

mPCOSQ Nederlands

Instructies: Deze vragenlijst is ontwikkeld voor vrouwen met Polycysteus Ovarium Syndroom. In de vragenlijst zullen we verwijzen naar het Polycysteus Ovarium Syndroom met de afkorting PCOS. De vragen gaan over uw gezondheid en problemen die te maken hebben met uw gezondheid. Beantwoord elke vraag door het vakje aan te kruisen met het antwoord dat het beste uw gevoel weergeeft. Voor elke vraag heeft u zeven antwoordmogelijkheden. Optie 1 vertegenwoordigt de grootst mogelijke last weer, terwijl optie 7 de minste last weergeeft. Kies slechts één antwoord voor elke vraag. Er zijn geen goede of foute antwoorden, kies het antwoord dat uw gevoel het beste weergeeft.

In welke mate heeft u het gevoel gehad dat zichtbare haargroei op uw kin een probleem voor u is geweest in de afgelopen twee weken:

	Een ernstig probleem	Een groot probleem	Een gemiddeld probleem	Enigszins een probleem	Een klein probleem	Nauwelijks een probleem	Geen probleem
1: Groei van zichtbaar haar op kin?	☐	☐	☐	☐	☐	☐	☐

Hoe vaak heeft u zich in de afgelopen twee weken ... gevoeld:

	De hele tijd	Het merendeel van de tijd	Redelijk deel van de tijd	Een deel van de tijd	Een klein deel van de tijd	Nauwelijks	Niet
2: Depressief als gevolg van het hebben van PCOS?	☐	☐	☐	☐	☐	☐	☐
3: Bezorgd over uw overgewicht?	☐	☐	☐	☐	☐	☐	☐
4: Snel vermoeid?	☐	☐	☐	☐	☐	☐	☐
5: Bezorgd over onvruchtsbaarheids-problemen?	☐	☐	☐	☐	☐	☐	☐
6: Humeurig als gevolg van het hebben van PCOS?	☐	☐	☐	☐	☐	☐	☐

In welke mate waren tijdens uw laatste menstruatie, de volgende zaken een probleem voor u:

	Een ernstig probleem	Een groot probleem	Een gemiddeld probleem	Enigszins een probleem	een klein probleem	Nauwelijks een probleem	Geen probleem
7: Hoofdpijn?	☐	☐	☐	☐	☐	☐	☐
8: Onregelmatige Menstruatie?	☐	☐	☐	☐	☐	☐	☐

In welke mate was zichtbare haargroei op uw bovenlip in de afgelopen twee weken een probleem voor u:

	Een ernstig probleem	Een groot probleem	Een gemiddeld probleem	Enigszins een probleem	Een klein probleem	Nauwelijks een probleem	Geen probleem
9: Groei van zichtbaar haar op uw bovenlip?	☐	☐	☐	☐	☐	☐	☐

Hoe vaak heeft u in de afgelopen twee weken:

	De hele tijd	Het merendeel van de tijd	Redelijk deel van de tijd	Een deel van de tijd	Een klein deel van de tijd	Nauwelijks	Niet
10: Moeite gehad met het accepteren van uw gewicht?	☐	☐	☐	☐	☐	☐	☐
11: Een negatief zelfbeeld gehad door het hebben van PCOS?	☐	☐	☐	☐	☐	☐	☐
12: Zich gefrustreerd gevoeld bij het proberen af te vallen?	☐	☐	☐	☐	☐	☐	☐
13: Bang geweest om geen kinderen te kunnen krijgen?	☐	☐	☐	☐	☐	☐	☐
14: Bang geweest om kanker te krijgen?	☐	☐	☐	☐	☐	☐	☐

In welke mate zijn de volgende zaken een probleem voor u geweest in de afgelopen twee weken:

	Een ernstig probleem	Een groot probleem	Een gemiddeld probleem	Enigszins een probleem	Een klein probleem	Nauwelijks een probleem	Geen probleem
15: Groei van zichtbaar haar op uw gezicht?	☐	☐	☐	☐	☐	☐	☐
16: Schaamte voor overtollig lichaamshaar?	☐	☐	☐	☐	☐	☐	☐

Hoe vaak heeft u zich in de afgelopen twee weken ... gevoeld:

	De hele tijd	Het merendeel van de tijd	Redelijk deel van de tijd	Een deel van de tijd	Een klein deel van de tijd	Nauwelijks	Niet
17: Bezorgd over het hebben van PCOS?	☐	☐	☐	☐	☐	☐	☐
18: Onzeker door het hebben van PCOS?	☐	☐	☐	☐	☐	☐	☐

In welke mate waren tijdens uw laatste menstruatie, de volgende zaken voor u een probleem:

	Een ernstig probleem	Een groot probleem	Een gemiddeld probleem	Enigszins een probleem	Een klein probleem	Nauwelijks een probleem	Geen probleem
19: Opgezwollen buik?	☐	☐	☐	☐	☐	☐	☐
20: Late menstruatie?	☐	☐	☐	☐	☐	☐	☐
21: Menstruatie-Krampen?	☐	☐	☐	☐	☐	☐	☐

Hoe vaak heeft/bent u in de afgelopen twee weken:

	De hele tijd	Het merendeel van de tijd	Redelijk deel van de tijd	Een deel van de tijd	Een klein deel van de tijd	Nauwelijks	Niet
22: Het gevoel gehad dat u niet sexy was door uw overgewicht?	☐	☐	☐	☐	☐	☐	☐
23: Een gebrek aan controle over de situatie met PCOS ervaren?	☐	☐	☐	☐	☐	☐	☐
24: Moeite gehad om op uw streefgewicht te blijven?	☐	☐	☐	☐	☐	☐	☐
25: Verdrietig geweest door problemen met uw vruchtbaarheid?	☐	☐	☐	☐	☐	☐	☐

In welke mate was zichtbaar lichaamshaar in de afgelopen twee weken een probleem voor u:

	Een ernstig probleem	Een groot probleem	Een gemiddeld probleem	Enigszins een probleem	Een klein probleem	Nauwelijks een probleem	Geen probleem
26: Groei van zichtbaar lichaams-haar?	☐	☐	☐	☐	☐	☐	☐

In welke mate zijn de volgende zaken een probleem voor u geweest in de afgelopen twee weken:

	Een ernstig probleem	Een groot probleem	Een gemiddeld probleem	Enigszins een probleem	Een klein probleem	Nauwelijks een probleem	Geen probleem
27: Acne (puistjes)?	☐	☐	☐	☐	☐	☐	☐

Hoe vaak heeft u zich in de afgelopen twee weken ... gevoeld:

	De hele tijd	Het merendeel van de tijd	Redelijk deel van de tijd	Een deel van de tijd	Een klein deel van de tijd	Nauwelijks	Niet
28: Onaantrekkelijk door het hebben van acne (puistjes)?	☐	☐	☐	☐	☐	☐	☐
29. Depressief door het hebben van acne (puistjes)?	☐	☐	☐	☐	☐	☐	☐

In welke mate was tijdens uw laatste menstruatie, ….. voor u een probleem:

	Een ernstig probleem	Een groot probleem	Een gemiddeld probleem	Enigszins een probleem	Een klein probleem	Nauwelijks een probleem	Geen probleem
30. Acne (puistjes)?	☐	☐	☐	☐	☐	☐	☐

Scoring (scoor elk antwoord van 1 tot 7):

Gewicht: 3 + 10 + 12 + 22 + 24/ 5

Lichaamsbeharing: 1 + 9 + 15 + 16 + 26 / 5

Emoties: 2 + 4 + 6 + 11 + 14 + 17 + 18 + 23 / 8

Acne: 27 + 28 + 29 + 30 / 4

Vruchtbaarheid: 5 + 13 + 25 / 3

Menstruatie problemen: 7 + 8 + 19 + 20 + 21 / 5

Een hogere score betekent een betere kwaliteit van leven.
