# Supplementary material for: Translation of the Modified Polycystic Ovary Syndrome Questionnaire (mPCOSQ) and the Polycystic Ovary Syndrome Quality of Life Tool (PCOSQOL) in Dutch and Flemish Women with PCOS
Source: J Clin Med. 2023 Jun 8;12(12):3927. doi: 10.3390/jcm12123927 (PMC10299068; doi:10.3390/jcm12123927)
Supplement: Supplementary file 1 [file jcm-12-03927-s001.zip › Supplementary File S2 Dutch version of the PCOSQOL.pdf]

PCOSQOL Nederlands

Deze vragenlijst is specifiek ontworpen om de kwaliteit van leven van vrouwen met polycysteus ovariumsyndroom (PCOS) te meten. De vragen in deze vragenlijst gaan over uw PCOS, uw gezondheid en problemen die te maken hebben met uw gezondheid. Lees en reageer op elke stelling door de optie te selecteren die aangeeft hoe u zich het meest voelde. Er zijn geen goede of foute antwoorden, u kiest gewoon het antwoord dat het beste weergeeft hoe u zich voelt. Als u vindt dat een vraag niet op u van toepassing is, selecteert u 'Niet van toepassing'.

Hoe vaak heeft/bent u in de afgelopen vier weken:

		Meestal	Vaak	Soms	Af en toe	Zelden	Helemaal niet	NVT
1	Het gevoel gehad dat u onder druk werd gezet om een kind te krijgen	1	2	3	4	5	6	7
2	Een schuldgevoel gehad omdat u overmatig agressief heeft gereageerd op een vriend(in) of familielid	1	2	3	4	5	6	7
3	Overdreven gereageerd op een dagelijkse gebeurtenis als gevolg van PCOS	1	2	3	4	5	6	7
4	Het gevoel gehad dat u geen echte vrouw was vanwege uw PCOS	1	2	3	4	5	6	7
5	Zich overweldigd gevoeld door uw PCOS en depressie	1	2	3	4	5	6	7
6	Zich depressief gevoeld over hoe PCOS van invloed is op uw leven	1	2	3	4	5	6	7
7	Zich zorgen gemaakt over dat andere mensen uw gezichtshaar kunnen zien	1	2	3	4	5	6	7
8	Bezorgd geweest om niet zwanger te kunnen worden	1	2	3	4	5	6	7
9	Zich depressief gevoeld over moeilijkheden om zwanger te worden	1	2	3	4	5	6	7
10	Zich minder vrouw gevoeld omdat u PCOS heeft	1	2	3	4	5	6	7
11	Ongeduldig geweest naar uw goede vrienden en/of familieleden toe	1	2	3	4	5	6	7
12	Het gevoel gehad dat u zonder reden wilde huilen	1	2	3	4	5	6	7
13	Iets willen doen maar het niet gedaan vanwege uw PCOS	1	2	3	4	5	6	7
14	Zich depressief gevoeld	1	2	3	4	5	6	7
15	Zich abnormaal gevoeld vanwege uw PCOS	1	2	3	4	5	6	7

		Meestal	Vaak	Soms	Af en toe	Zelden	Helemaal niet	Niet van toepassing
16	Het gevoel gehad dat u niet wist wat u moest doen om uzelf te helpen	1	2	3	4	5	6	7
17	Het gevoel gehad dat u niet wist wat u moest doen om uw PCOS te beheersen	1	2	3	4	5	6	7
18	Het gevoel gehad dat u uzelf haatte	1	2	3	4	5	6	7
19	Het gevoel gehad dat PCOS uw leven beheerste	1	2	3	4	5	6	7
20	Zich geschaamd over hoe u eruit zag	1	2	3	4	5	6	7
21	Zich humeurig gevoeld vanwege uw overtollige haargroei	1	2	3	4	5	6	7
22	Zich depressief gevoeld vanwege uw overtollige haargroei	1	2	3	4	5	6	7
23	Zich depressief gevoeld als gevolg van uw onvruchtbaarheid	1	2	3	4	5	6	7
24	Veel tijd en energie besteed aan het verwijderen van overtollig haar	1	2	3	4	5	6	7
25	Zich depressief gevoeld over uw strijd om kinderen te krijgen	1	2	3	4	5	6	7
26	Zich depressief gevoeld vanwege uw gezichtshaar	1	2	3	4	5	6	7
27	Zich boos gevoeld omdat u PCOS heeft	1	2	3	4	5	6	7
28	Jaloers geweest op vrouwen zonder PCOS	1	2	3	4	5	6	7
29	Zich geschaamd omdat u PCOS heeft	1	2	3	4	5	6	7
30	Zich geschaamd voor uw gezichtshaar	1	2	3	4	5	6	7
31	Zich een mislukking gevoeld omdat u moeite heeft met zwanger worden	1	2	3	4	5	6	7
32	Het gevoel gehad dat het oneerlijk is dat u PCOS heeft	1	2	3	4	5	6	7
33	Moeite gehad te leren omgaan met uw PCOS en uw andere aandoening(en)	1	2	3	4	5	6	7
34	Bang geweest dat u mogelijk geen kinderen zou kunnen krijgen	1	2	3	4	5	6	7
35	Deel willen nemen aan een sociale activiteit, maar het niet gedaan vanwege uw PCOS	1	2	3	4	5	6	7

Scoring (scoor elk antwoord van 1 tot 7):

Impact van PCOS:	4 + 5 + 6 + 10 + 13 + 14 + 15 + 16 + 17 + 18 + 35 / 11
Vruchtbaarheid	1 + 8 + 9 + 23 + 25 + 31 + 34 / 7
Overbeharing	7 + 20 + 21 + 22 + 24 + 26 + 30 / 6
Omgaan met PCOS	19 + 27 + 28 + 29 + 32 + 33 / 6
Stemming	2 + 3 + 11 + 12 / 4

Een hogere score betekent een betere kwaliteit van leven.
