# Supplementary material for: Translation of the Modified Polycystic Ovary Syndrome Questionnaire (mPCOSQ) and the Polycystic Ovary Syndrome Quality of Life Tool (PCOSQOL) in Dutch and Flemish Women with PCOS
Source: J Clin Med. 2023 Jun 8;12(12):3927. doi: 10.3390/jcm12123927 (PMC10299068; doi:10.3390/jcm12123927)
Supplement: Supplementary file 1 [file jcm-12-03927-s001.zip › Supplementary Tables 1 and 2.pdf]

Supplemental tables

Supplemental Table 1: mPCOSQ factor analysis

	Item		1	2	3	4	5	6
**Weight**	3	Concerned about being overweight?	0.904					
	10	Trouble dealing with weight?	0.913					
	12	Frustration in trying to lose weight?	0.911					
	22	Felt unsexy because of overweight?	0.879					
	24	Difficulties staying at your ideal weight?	0.911					
**Body hair**	1	Growth of visible hair on chin?		0.862				
	9	Growth of visible hair on upper lip?		0.812				
	15	Growth of visible hair on your face?		0.917				
	16	Embarrassment about excessive body hair?		0.852				
	26	Growth of visible body hair?		0.892				
**Emotions**	2	Depressed as a result of having PCOS?			0.687			
	4	Tired easily?			0.537			
	6	Moody as a result of having PCOS?			0.701			
	11	Low self-esteem as a result of having PCOS?			0.648			
	14	Afraid of getting cancer?			0.597			
	17	Worried about having PCOS?			0.656			
	18	Self-conscious as a result of having PCOS?			0.686			
	23	Lack of control over the situation with PCOS?			0.592			
**Acne**	27	Acne (last two weeks)?				0.924		
	28	Felt unattractive because of acne?				0.948		
	29	Depressed as a result of acne?				0.834		
	30	Acne (in relation to last menstruation)?				0.888		
**Infertility**	5	Concerned about infertility problems?					0.899	
	13	Afraid of not being able to have children?					0.834	
	25	Sad because of infertility problems?					0.870	
**Menstrual problems**	7	Headaches?						0.608
	8	Irregular menstrual periods?						0.719
	19	Abdominal bloating?						0.651
	20	Late menstrual period?						0.694

	21	Menstrual cramps?	0.600

Supplemental Table 2: PCOSQOL factor analysis

Factor	Item		1	2	3	4	5
**Impact of PCOS**	4	Felt like you weren't a real woman because of your PCOS	0.545				
	5	Felt overwhelmed by your PCOS and depression	0.801				
	6	Felt depressed about how PCOS has impacted your life	0.767				
	10	Felt like less of a woman because of having PCOS	0.590				
	13	Wanted to do something but haven't because of your PCOS	0.673				
	14	Felt depressed	0.739				
	15	Felt abnormal because of your PCOS	0.678				
	16	Felt like you don't know what to do to help yourself	0.610				
	17	Felt like you don't know what to do to control your PCOS	0.543				
	18	Felt like you hated yourself	0.597				
	35	Wanted to take part in a social activity but haven't because of your PCOS	0.649				
**Infertility**	1	Felt under pressure to have a child		0.661			
	8	Felt anxious about conceiving a child		0.905			
	9	Felt depressed over difficulties conceiving a child		0.880			
	23	Felt depressed because of your infertility		0.860			
	25	Felt depressed over your struggle to have children		0.878			
	31	Felt like a failure because of your trouble conceiving		0.827			
	34	Been scared that you may not have children		0.852			
**Hirsutism**	7	Been worried about other people seeing your facial hair			0.880		
	20	Felt embarrassed about the way you look			*<0.500*		
	21	Felt moody because of your excess hair			0.861		
	22	Felt depressed because of your hirsutism			0.796		
	24	Spent a lot of time and energy removing excess hair			0.854		
	26	Felt depressed because of your facial hair			0.830		
	30	Felt embarrassed by your facial hair			0.900		
**Coping with PCOS**	19	Felt like your PCOS is in control of your life	0.503			0.558	
	27	Felt angry that you have PCOS				0.673	
	28	Been envious of women without PCOS				0.797	

	29	Felt embarrassed about having PCOS	0.561
	32	Felt that it is unfair that you have PCOS	0.724
	33	Struggled to cope with your PCOS and your other condition(s)	0.639
**Mood**	2	Felt guilty for being overly aggressive towards a friend or family member	0.793
	3	Over reacted to a day-to-day occurrence because of your PCOS	0.632
	11	Had a short temper with your close friends and/or family	0.803
	12	Felt like crying for no reason	0.647
